# Keeping Green and Functional: Photosynthetic Integrity and Leaf Area Underpin Waterlogging Tolerance in Bread Wheat

**DOI:** 10.3390/plants15131995

**Published:** 2026-06-27

**Authors:** Isabel P. Pais, José N. Semedo, Paula Scotti-Campos, Cláudia C. Pessoa, Fernando C. Lidon, Benvindo Maçãs, José C. Ramalho

**Affiliations:** 1 Unit for Research and Services in Biotechnology and Genetic Resources, National Institute of Agricultural and Veterinarian Research, Quinta do Marquês, Av. da República, 2780-157 Oeiras, Portugal; jose.semedo@iniav.pt (J.N.S.); paula.scotti@iniav.pt (P.S.-C.); 2 GeoBioSciences, GeoTechnologies and GeoEngineering Unit (GeoBioTec), Department of Earth Sciences, Faculty of Sciences and Technology, NOVA University of Lisbon, 2829-516 Caparica, Portugal; c.pessoa@fct.unl.pt (C.C.P.); fjl@fct.unl.pt (F.C.L.); 3 Unit for Research and Services in Biotechnology and Genetic Resources, National Institute of Agricultural and Veterinarian Research, Estrada de Gil Vaz, 7351-901 Elvas, Portugal; benvindomacas@gmail.com; 4 Plant Stress Ecophysiology and Biochemistry Laboratory, Forest Research Center (CEF), Associate Laboratory TERRA, School of Agriculture (ISA), University of Lisbon (ULisboa), Quinta do Marquês, Av. da República, 2784-505 Oeiras, Portugal; cochichor@mail.telepac.pt

**Keywords:** genotypic variability, leaf gas exchanges, leaf senescence, morphological traits, tillering stage, *Triticum aestivum* L., flooding

## Abstract

Waterlogging at the tillering stage, a key early vegetative growth stage, is increasingly limiting wheat productivity worldwide, but the physiological mechanisms underlying genotypic tolerance are not fully understood. To address this, 23 bread wheat (*Triticum aestivum* L.) genotypes from five germplasm groups were exposed to 14 days of waterlogging at the tillering stage. Morphological traits including leaf (green area, biomass, and senescent biomass proportion) and the elongation rate of the main culm (cm day^−1^), plant water status (relative water content, RWC), photosynthetic pigment content (SPAD values; total chlorophyll, TChl; total carotenoids, TCar), and photosynthetic performance (maximal photochemical efficiency of photosystem II, F_v_/Fm; actual photochemical efficiency of photosystem II, F_v_′/F_m_′; net photosyntheis, P_n_; stomatal conductance to water vapor, g_s_), were assessed. Waterlogging induced strong but highly variable responses among genotypes. Sensitive genotypes showed marked reductions in green biomass (up to ~40–60%), TChl content (up to ~80%), TCar (~70%), and photosynthetic performance, including declines in F_v_/F_m_, F_v_′/F_m_′, and P_n_. In contrast, tolerant genotypes maintained higher photochemical efficiency, P_n_, and pigment content, despite stress exposure, underscoring greater functional resilience. Importantly, morphological stability did not consistently translate into functional performance. Several genotypes maintained green leaf area despite pronounced declines in photosynthetic capacity and pigment content, revealing a decoupling between morphological and physiological responses. Multivariate analysis identified an integrated photosynthetic trait axis strongly associated with yield performance under stress, highlighting that tolerance is primarily driven by the capacity to maintain photosynthetic function rather than green biomass alone. Together, these findings emphasize the importance of preserving both physiological functionality and green leaf area to maintain waterlogging tolerance. Integrated physiological markers (e.g., TChl and TCar content, photochemical quenching, leaf gas exchange traits) enable effective early screening and support function-based selection in wheat breeding programs.

## 1. Introduction

The management of plant resources has been fundamental to human societies, with wheat domestication from early diploid and tetraploid species to the later emergence of hexaploid *Triticum aestivum* L. ca. 10,000 years ago, a key step in the development and global spread of agriculture [1,2,3,4]. Nowadays, wheat is one of the most widely cultivated food crops worldwide, with an annual production of ca. 800 million metric tons, of which Europe contributes to ca. 30.5% [5].

Wheat broad environmental adaptability, partly attributed to its hexaploidy, and its major contribution to human caloric and protein intake led it to play a crucial role in global food security and nutrition [4,6,7] for a growing human population. Yet, despite significant advancements in breeding and agronomic practices, wheat productivity remains largely dependent on climatic conditions. In fact, climate change is increasingly affecting agricultural productivity and quality, namely, in wheat, largely due to a greater frequency and severity of stress events of water (drought, waterlogging, and flooding) and extreme temperatures (heat, cold), as well as soil degradation due to salinization [8,9,10,11,12,13,14,15]. Recent evidence shows that global wheat yields declined by ca. 8 to 12% over the last four decades, highlighting the sensitivity of this crop to a quick change in agroclimatic conditions [16,17,18]. Projections further suggest that negative impacts will intensify, particularly in vulnerable regions, such as the Mediterranean basin, where the frequency and severity of extreme climatic events are anticipated to rise and where yield reductions of up to 60% have been projected under future climate scenarios [19,20]. This region, which includes the Iberian Peninsula, is known as a climate change hotspot and has already experienced increasingly erratic rainfall patterns, with longer dry periods interspersed with intense rainfall events, with direct consequences for wheat production in recent years [21].

While drought and heat stress impacts have been thoroughly studied in wheat [22,23,24,25,26], the impacts of excessive soil moisture have received relatively little attention, despite its increasing relevance. The rising occurrence of severe precipitation events has resulted in more frequent episodes of waterlogging (and even flooding), exposing crops to hypoxic or anoxic conditions. Under these rhizosphere environments, root respiration, nutrient absorption, and energy metabolism are severely impaired, triggering a cascade of physiological responses, including stomatal closure and photosynthesis decline linked to dysfunction of the photosynthetic apparatus (e.g., Chl degradation, PS II inhibition), and accelerated leaf senescence, ultimately compromising the preservation of functional green leaf area [27,28]. Although there is an increasing wealth of literature regarding waterlogging impact in cereals, important knowledge gaps remain. In fact, most studies have focused on end-point traits, such as yield or total plant biomass, often overlooking early mechanistically physiological indicators that could support genotype screening to this stress. Also, single-trait approaches dominate, failing to capture the integrative nature of plant responses to hypoxia. This limitation is particularly evident in *T. aestivum*, where intraspecific variability during the tillering stage, a critical phase for yield determination, remains poorly understood [3,11,12,29,30].

Our previous work has emphasized the need for comprehensive, multivariate assessments to link physiological performance with morphological traits and early stress responses [11,29,30]. Supported by these findings, the present study applies an integrated ecophysiological and morphological approach to evaluate a diverse panel of bread wheat genotypes under controlled waterlogging during the tillering stage. By simultaneously assessing photosynthetic performance, plant water status, pigment content, and green leaf area, we aim to identify key traits that reliably differentiate tolerant and sensitive genotypes, addressing a current knowledge gap in early-stage screening while providing practical indicators/tools that can be directly used in breeding programs to enhance wheat resilience to waterlogging.

## 2. Results

Waterlogging during the tillering stage induced physiological and morphological changes across the twenty-three bread wheat genotypes evaluated. The extent and nature of the response varied markedly among genotypes, indicating different degrees of tolerance to excessive soil moisture. In the following sections, and after confirmation of hypoxic conditions based on soil redox potential, the results are presented according to categories: (i) morphological and water status traits; (ii) pigment content and Chl status; (iii) photosynthetic performance and gas exchange; and (iv) integrated multivariate analysis. This approach enables a comprehensive assessment of key traits underlying stress tolerance/sensitivity, identifying genotypes capable of maintaining both green leaf structure and photosynthetic functioning under waterlogging.

### 2.1. Soil Redox Potential as an Indicator of Hypoxic Conditions

No differences in soil redox potential (Eh) were observed between well-watered (WW) and waterlogged (WL) pots immediately prior to the imposition of waterlogging (T0) (Table 1). In the WW treatment, Eh remained stable throughout the experimental period, with average values of approximately 400 mV, a range considered favorable for plant development (Table 1). In contrast, waterlogging induced a rapid decline in Eh within 24 h of stress imposition (T1), reflecting the onset of oxygen depletion in the root zone. Thereafter, Eh values in WL pots remained significantly lower than those recorded in WW pots and continued to decrease throughout the experimental period. By the 14th day of waterlogging, Eh reached negative values (−11.9 mV), indicating strongly reduced soil conditions and the establishment of an anaerobic environment (Table 1).

### 2.2. Morphological and Hydric Responses Under Waterlogging

Waterlogging significantly affected plant structure and water status in a genotype-dependent manner, considering the main culm elongation rate, green leaf area, foliar biomass, proportion of senescent leaf biomass, and relative water content (RWC).

#### 2.2.1. Elongation Rate of the Main Culm

**Figure 1:**
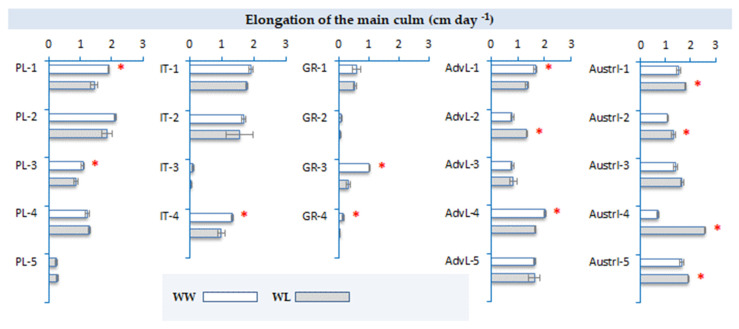
Daily elongation rate of the main culm (cm day^−1^) during the 14-day waterlogging period in *T. aestivum* L. genotypes from five germplasm groups with distinct genetic backgrounds: Portuguese landraces (PL); varieties with introduced Italian germplasm (IT); Post-Green Revolution varieties with introduced CIMMYT germplasm (GR); advanced lines (AdvL) from the Portuguese Cereal Breeding Programme; and Australian varieties (Austrl). For each genotype, differences between water regimes (WW and WL) were assessed by one-way ANOVA (*n* = 3–6, *p* < 0.05). * Indicates significant differences between WW and WL placed on the higher mean value for each genotype.

A wide range of daily elongation rates was observed among the studied genotypes under both water regimes. Under well-watered conditions (WW), main culm elongation rates ranged from 0.07 to 2.1 cm day^−1^ (GR-2 and PL-2, respectively) (Figure 1).

Waterlogging further increased this variability, resulting in values from 0.04 (GR-4) to 2.6 cm day^−1^ (Austrl-4) (Figure 1). In fact, contrasting responses were found under WL, with seven genotypes showing a significant decline in their elongation rates, to less than half in some cases (IT-3, GR-3, and GR-4), indicating strong growth inhibition. A second group of 11 genotypes showed no significant impact on this parameter. In contrast, five genotypes, four of which belong to the Australian germplasm, displayed increased elongation rates under WL. Among them, Austrl-4 stood out, due to an increase of 2.7-fold of their elongation rate, from 0.7 (WW) to 2.6 (WL) cm day^−1^.

#### 2.2.2. Waterlogging-Induced Changes in Foliar Biomass and Leaf Senescence

**Figure 2:**
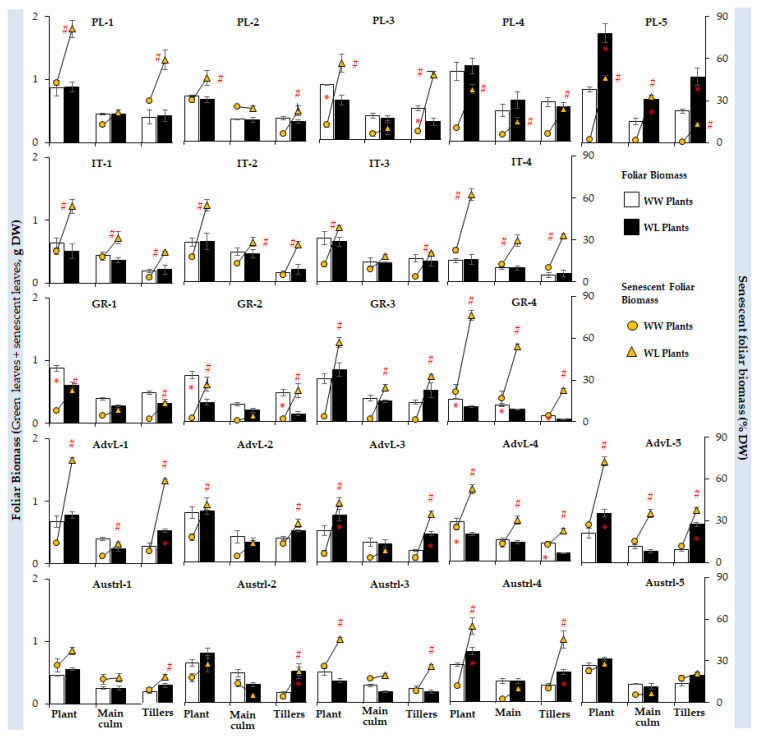
Total leaf biomass (g DW) and senescent leaf biomass (% DW) in *T. aestivum* L. genotypes from five germplasm groups with distinct genetic backgrounds: Portuguese landraces (PL), varieties with introduced Italian germplasm (IT), Post-Green Revolution varieties with CIMMYT germplasm (GR), advanced lines (AdvL) from the Portuguese Cereal Breeding Programme, and Australian varieties (Austrl). Measurements were performed after 14 days of treatment (T14) under well-watered (WW) and waterlogged (WL) conditions. Bars (left axis) represent total leaf dry biomass (green + senescent leaves, g DW) of the whole plant, main culm, and tillers, whereas lines (right axis) represent the proportion of senescent leaf biomass (% DW) for the same organs. Statistical analyses were performed separately for total and senescent leaf biomass for each genotype using two-way ANOVA followed by Tukey’s HSD test (*n* = 3, *p* < 0.05). Significant differences between WW and WL are indicated by * for total leaf biomass and **#** for senescent leaf biomass, shown above the higher mean value.

Evaluations performed at the 14th day of waterlogging showed that foliar biomass (plant, g DW) in WL plants, when compared with WW plants at the same time point, was similar in 14 genotypes, whereas nine genotypes showed marked changes (four increases and five decreases) (Figure 2, bars). No significant changes were observed in PL-1, PL-2, PL-4, all IT genotypes, GR-3, AdvL-2, Austrl-1, Austrl-3, and Austrl-5, either at the whole plant level or when the main culm and tillers were evaluated separately. Additionally, AdvL-1 and Austrl-2 also maintained foliar biomass at the plant level, although this was achieved by increased tiller biomass that compensated for the tendency to decline in the main culm (Figure 2, bars).

WL promoted reductions in foliar biomass (25.9–58.4%) in PL-3, GR-2, and AdvL-4, mainly due to tiller biomass declines (42.8–72.2%). In GR-4, decreased plant foliar biomass resulted from declines in the main culm (26.7%) and tillers (51.0%), whereas in GR-1, plant foliar biomass resulted from non-significant changes in both organs. Interestingly, WL raised plant foliar biomass in PL-5, AdvL-3, AdvL-5, and Austrl-4. In PL-5, biomass more than doubled in both the main culm (103.7%) and tillers (108.1%), while in AdvL-3, AdvL-5, and Austrl-4, increases were mainly associated with tillers, which rose by 79.7–189.5% (Figure 2, bars).

In WW plants, the proportion of senescent leaf biomass (i.e., the percentage of total foliar biomass represented by senescent leaves in the whole plant, main culm, and tillers), assessed at the end of the 14-day WL period, ranged from 2.5% (PL-5) to 42.5% (PL-1) (Figure 2, lines). As WW plants were maintained under optimal irrigation, this variation reflects developmental (age-dependent) leaf senescence occurring during the experimental period. The higher values observed in some genotypes reflect the early senescence of basal leaves in the main culm, a pattern commonly reported in wheat even under optimal water supply.

With the WL implementation, the percentage of senescent leaf biomass increased significantly relative to WW in most genotypes, whereas Austrl-1, Austrl-2, and Austrl-5 showed only minor, non-significant rises (Figure 2, lines). The WL-induced increase (ΔWL–WW) ranged from 15.2 (PL-2) to 53.5 (GR-3) percentage points, showing genotypic variation in response magnitude. For example, foliar senescence in PL-1 increased from 42.5% to 82% (39.5%, 1.9-fold), whereas PL-5 increased from 2.5% to 46.4% (43.9%, 18.6-fold). While ΔWL–WW describes the magnitude of the response to waterlogging, the proportion of senescent leaf biomass under WL represents the final canopy status at the end of the stress period. Accordingly, Figure 2 presents the proportion of senescent values under both WW and WL, as this metric reflects canopy functional status, allowing direct comparison among genotypes. By the end of the 14-day stress period, plant senescent leaf biomass under WL ranged from 24 to 38% in the least affected (GR-1, GR-2, PL-4, and AdvL-3) to 70–82% in the most impacted ones (PL-1, GR-4, and AdvL-1). Several additional genotypes also reached high levels of senescence (>55%), including PL-3 (57.9%), IT-4 (61.5%), GR-3 (57.0%), and IT-1 (55.1%).

Senescence under WL was often more pronounced in tillers than in the main culm, as observed in PL-2, PL-3, PL-4, IT-2, IT-3, IT-4, GR-2, AdvL-1, AdvL-3, Austrl-1, Austrl-2, and Austrl-4 (12 genotypes), reflecting differential distribution of senescence within the plant. A similar extent of senescence between tillers and the main culm was found in PL-1, IT-1, GR-1, GR-3, AdvL-2, AdvL-5, Austrl-3, and Austrl-5 (eight genotypes), while only three genotypes were more impacted in the main culm (PL-5, GR-4, AdvL-4). Only AdvL-4, Austrl-1, and Austrl-5 maintained similar senescent levels (%) in tillers under WL compared with their WW plants. In contrast, twelve genotypes (PL-2, PL-4, IT-2, IT-3, GR-2, AdvL-1, AdvL-2, AdvL-3, and all Australians except Austrl-3) exhibited stable senescence of the MC biomass under WL (Figure 2, lines).

**Figure 3:**
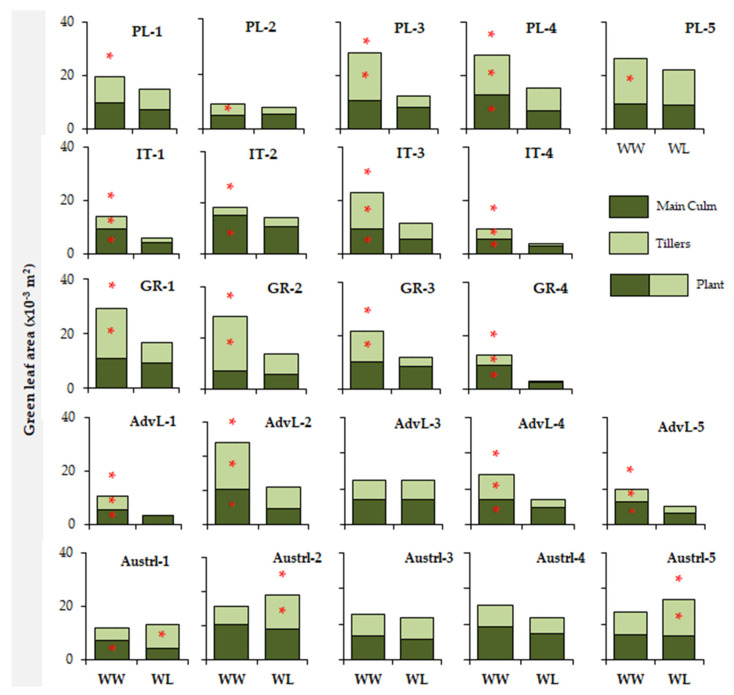
Green leaf area (×10^−3^ m^2^) in *T. aestivum* L. genotypes from five germplasm groups with distinct genetic backgrounds: Portuguese landraces (PL), varieties with the introduction of Italian germplasm (IT), Post-Green Revolution varieties with CIMMYT germplasm (GR), advanced lines (AdvL) from the Portuguese Cereal Breeding Programme, and Australian varieties (Austrl). Measurements were performed after 14 days of waterlogging under well-watered (WW) and waterlogged (WL) conditions. Bars show total plant green leaf area (MC + T), with dark green representing the main culm (MC) and light green representing total tillers (T). Asterisks (*) indicate statistically significant differences between WL and WW for total plant area, main culm, or tillers at T14, placed at the higher mean value. Statistical analysis was performed per genotype using two-way ANOVA followed by Tukey’s HSD test (*n* = 3–6, *p* < 0.05; simple effects of WW vs. WL are shown).

Distinct responses to WL emerged regarding the relative contribution of the main culm and/or tillers to plant green leaf area (Figure 3). The genotypes AdvL-3, Austrl-3, and Austrl-4 showed remarkable morphological stability, with unchanged green area across both components. Another group included highly impacted genotypes (PL-4, IT-1, IT-3, IT-4, GR-4, AdvL-1, AdvL-2, AdvL-4, and AdvL-5), which exhibited coordinated reductions in both main culm (34.5–70.8%) and tillers (42.6–100%), resulting in heavy losses of total green area (45.6–78.5%), thus indicating generalized morphological impairments promoted by WL (Figure 3). PL-3, GR-1, GR-2, and GR-3 displayed a tiller-dominant reduction pattern, with declines (43.3–51.1%) in total green area being primarily driven by strong decreases in tiller area (60.1–70.9%), while the main culm showed more stable values. Finally, a subset of genotypes maintained (Austrl-1) or even increased (Austrl-2 and Austrl-5) total green area through stimulation of tiller growth, which in Austrl-1 likely compensated for the reduction observed in the main culm area (Figure 3). Overall, tiller green area showed greater variation than the main culm across genotypes. Morphological differences among genotypes indicated that maintenance or compensation of total green area was mainly associated with changes in tiller growth.

Two main response patterns emerged when relating senescence to changes in green leaf area under WL. A first group of genotypes showed increased senescence (Figure 2, Lines) associated with reductions in green leaf area (Figure 3). This group included PL-1, PL-3, PL-4, all IT and GR genotypes, and AdvL-1, AdvL-2, AdvL-4, and AdvL-5, with some also exhibiting reductions in total foliar biomass (Figure 2, bars). A second group maintained green leaf area despite increased senescence, including PL-2, PL-5, Austrl-3, Austrl-4, and AdvL-3. Except for the latter genotype, which showed consistently stable values, this apparent stability resulted from non-significant decreases in green leaf area, while foliar biomass was either stable or increased depending on the genotype.

#### 2.2.3. Leaf Water Status Under Waterlogging

**Figure 4:**
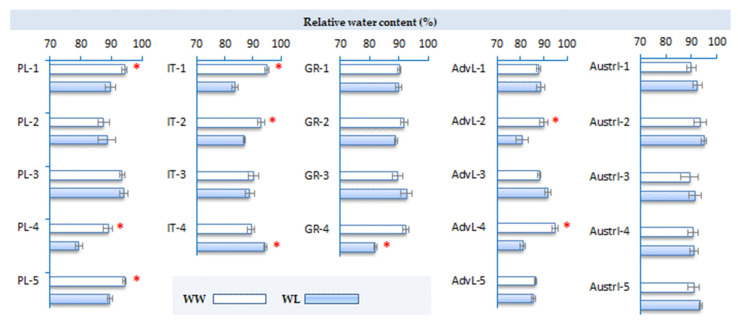
Leaf relative water content (RWC, %) in *T. aestivum* L. genotypes from five germplasm groups with distinct genetic backgrounds: Portuguese landraces (PL), varieties with introduced Italian germplasm (IT), Post-Green Revolution varieties with CIMMYT germplasm (GR), advanced lines (AdvL) from the Portuguese Cereal Breeding Programme, and Australian varieties (Austrl). Measurements were performed after 14 days of waterlogging in the youngest fully expanded leaf of the main culm from well-watered (WW) and waterlogged (WL) plants. For each genotype, differences between water regimes (WW, WL) are indicated with * (one-way ANOVA, *n* = 3, *p* < 0.05).

The maintenance of leaf hydration is closely associated with the preservation of functional leaf structure. Accordingly, relative water content (RWC) was determined as an indicator of leaf water status under control (WW) and waterlogging (WL) conditions, allowing the evaluation of the ability of genotypes to maintain cellular hydration under hypoxic stress and linking leaf structural integrity with downstream biochemical and photosynthetic processes (Figure 4).

Under WW conditions, most genotypes exhibited high RWC values (90–95%), indicating adequate leaf hydration. WL had little effect on leaf hydration in most genotypes. A large set of 14 genotypes (PL-2, PL-3, IT-3, GR-1, GR-2, GR-3, AdvL-1, AdvL-3, AdvL-5, and Australian varieties) was unaffected by stress, maintaining RWC values above 88% (Figure 4). Likewise, despite significant RWC reductions (PL-1, PL-5, and IT-2) or increases (IT-4), values remained ca. 90%, pointing to adequate leaf water status. In contrast, PL-4, IT-1, GR-4, AdvL-2, and AdvL-4 showed marked reductions under WL as compared to WW counterparts, with RWC values declining from 90–95% in WW to ca. 80%.

### 2.3. Leaf Pigment Characterization

#### 2.3.1. Chlorophyll Status (SPAD)

**Figure 5:**
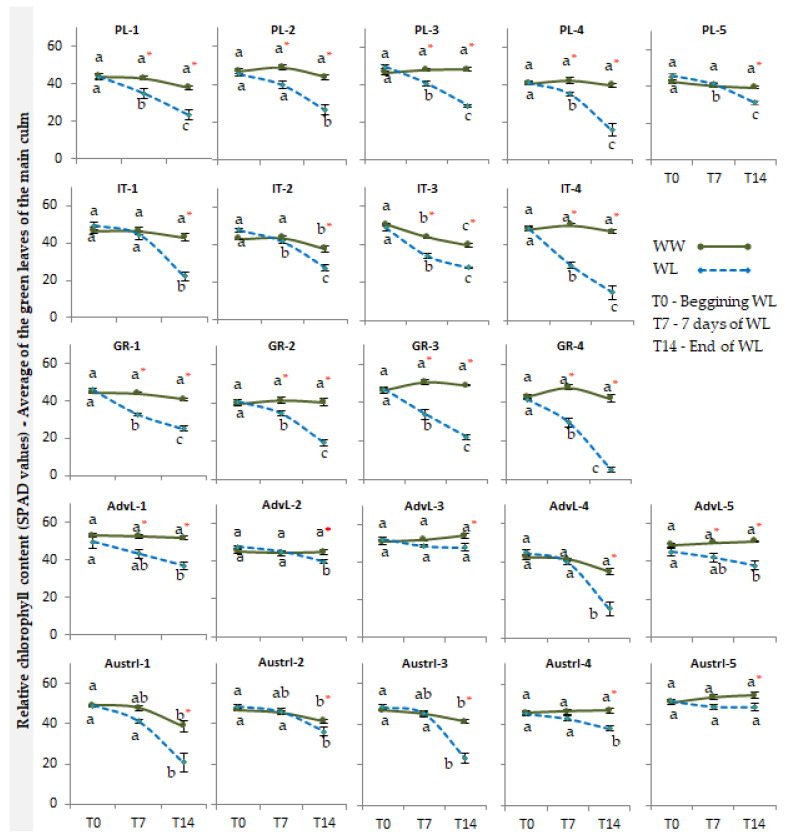
Relative chlorophyll content (SPAD) in *T. aestivum* L. genotypes from five germplasm groups with distinct genetic backgrounds: Portuguese landraces (PL), varieties with introduced Italian germplasm (IT), Post-Green Revolution varieties with CIMMYT germplasm (GR), advanced lines (AdvL) from the Portuguese Cereal Breeding Programme, and Australian varieties (Austrl). Measurements were performed under well-watered (WW) and waterlogged (WL) conditions at the beginning (T0), after 7 days (T7), and after 14 days of stress (T14). SPAD values represent the mean relative chlorophyll content of all green leaves from the main culm. For each genotype, different lowercase letters (a, b, c) indicate significant differences among sampling times (T0, T7, T14) within the same water regime, while * indicates significant differences between water regimes (WW vs. WL) at the same time point. In all cases, “a” and * denote the higher mean value. Statistical analysis was performed by two-way ANOVA followed by Tukey’s test (*n* = 3–6, *p* < 0.05).

Relative Chl status was monitored non-destructively using SPAD measurements (mean of all leaves from the main culm) at the beginning of the experiment, before WL implementation (T0), and after 7 (T7) and 14 (T14) days of WL exposure (Figure 5).

Starting from similar SPAD values at T0 (indicating similar Chl status), changes were found along the 14-day period, mainly under WL conditions. In WW plants, SPAD values remained relatively stable throughout the evaluation period in most genotypes. Still, gradual declines were observed in some cases, with significant reductions by T14 in IT-2 (12.5%), IT-3 (21.6%), Austrl-1 (21.3%), Austrl-2 (11.8%), and Austrl-3 (12.3%), likely reflecting normal developmental progression (Figure 5). Under WL, these genotypes maintained a similar declining trend, although with substantially greater reductions relative to T0, reaching 42.9%, 43.3%, 57.5%, 25.4%, and 52.2%, respectively. Overall, WL promoted a clear decline in SPAD values across all genotypes, although with variable magnitude and timing (Figure 5). Early reductions (T7) were evident in PL-1 to PL-4, IT-3, IT-4, GR-1 to GR-4, and AdvL-1 and AdvL-5, with decreases between 15 and 42%, increasing to 25–88% by T14. A second group showed a delayed response to WL, maintaining SPAD values until T7 but declining thereafter. This pattern was observed in PL-5, IT-1, IT-2, AdvL-2 to AdvL-4, and all Australian genotypes, with reductions of ca. 11–56% by T14 (Figure 5). By the end of the stress period, SPAD reductions under WL ranged widely (11–88%), with the most pronounced decreases observed in GR-4, IT-4, PL-4, and AdvL-4 (Figure 5).

#### 2.3.2. Total Chlorophylls and Carotenoids

**Figure 6:**
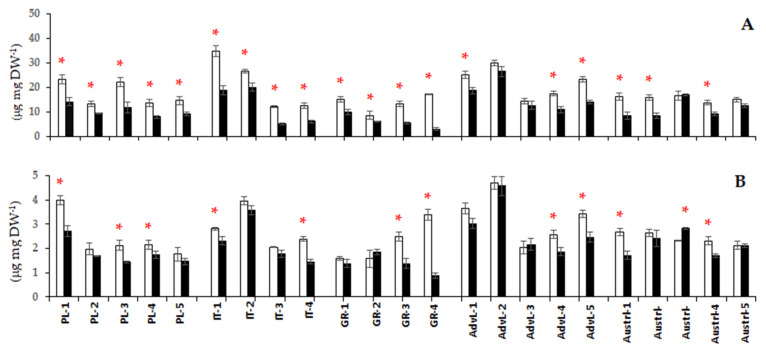
Total chlorophyll (**A**) and total carotenoid (**B**) content in well-watered (□) and waterlogged (■) plants after 14 days of stress. Quantifications (mg g^−1^ DW) were conducted on the same leaves used for gas exchange and chlorophyll fluorescence analyses in *T. aestivum* L. genotypes from five germplasm groups with distinct genetic backgrounds: Portuguese landraces (PL), varieties with introduced Italian germplasm (IT), Post-Green Revolution varieties with CIMMYT germplasm (GR), advanced lines (AdvL) from the Portuguese Cereal Breeding Programme, and Australian varieties (Austrl). Statistical analyses were performed separately for each genotype and trait using one-way ANOVA (*n* = 3, *p* < 0.05). Significant differences between water regimes (WW vs. WL) are indicated only when present, with * placed at the higher mean value.

Spectrophotometric measurements showed that the total chlorophyll (TChl) content decreased in WL plants of most genotypes by T14, with reductions ranging from 24.7 (IT-2) to 82.4%. The most pronounced declines were found in GR-4 (82.4%), GR-3 (58.8%), IT-3 (57.5%), and IT-4 (52.0%), while intermediate reductions (27.5–47.3%) occurred in the PL genotypes, as well as in several AdvL and Austrl genotypes. In contrast, AdvL-2, AdvL-3, Austrl-3, and Austrl-5 maintained stable TChl levels under WL (Figure 6A).

Regarding the response of total carotenoids (TCar) to WL, several genotypes (PL-2, PL-5, IT-2, IT-3, GR-1, GR-2, AdvL-1 to AdvL-3, Austrl-2, and Austrl-5) showed stable values, suggesting preservation of photoprotective pigment pools. In the remaining genotypes, TCar declined (18 to 73.9%), with the strongest decreases observed in GR-4 (73.9%), GR-3 (44.8%), IT-4 (39.0%), and Austrl-1 (35.7%) (Figure 6B). Notably, Austrl-3 was the only genotype to show a significant rise of TCar under WL.

### 2.4. Leaf Gas Exchanges and Photosystem II Photochemical Efficiency

The ability to maintain photosynthetic activity under WL conditions is a key trait associated with stress tolerance in bread wheat. Photosynthetic performance was assessed through maximal (F_v_/F_m_, dark-adapted) and actual (F_v_′/F_m_′, photosynthetic steady-state) photochemical efficiency of photosystem II (PSII), as well as leaf gas exchange parameters (net photosynthesis, P_n_, and stomatal conductance to water vapor, g_s_), reflecting the efficiency of photochemical processes and gas exchange regulation (Table 2).

In WW plants, F_v_/F_m_ values ranged from 0.798 to 0.824, which is typical for healthy wheat leaves. Under WL, most genotypes preserved values close to those of control plants, even if presenting a significant difference to WW plants. In fact, the significant changes found in PL-1, PL-2, Austrl-4, and Autrl-5 were small (≤4%), with F_v_/F_m_ values remaining close to those of WW plants and within a range typically associated with intact PSII reaction centers, whereas a smaller group of genotypes exhibited more pronounced reductions, such as Austrl-3 (11.8%) and IT-1 (8.1%). Sharp declines were observed in GR-4 and AdvL-4, where F_v_/F_m_ declined by 71.5% and 35.2%, reaching values of 0.229 and 0.528, respectively, suggesting severe impairment of PSII photochemistry (Table 2). F_v_′/F_m_′ exhibited greater variability with several genotypes showing moderate reductions under WL, with others maintaining similar values or even exhibiting increases (IT-3, GR-3, and Austrl-5), whereas marked declines were recorded in IT-1, AdvL-4, and Austrl-3. Still, similarly to F_v_/F_m_ findings, the greatest impacts were also found in GR-4 and AdvL-4, which showed declines of 80% and 57% in F_v_′/F_m_′, respectively.

Regarding leaf gas exchange parameters, the net photosynthetic rate (P_n_) was strongly affected by WL in almost all genotypes (Table 2). While in WW plants, P_n_ values ranged from 11.2 to 21.2 µmol CO_2_ m^−2^ s^−1^, in WL, a wider range (2.5–22.9 µmol CO_2_ m^−2^ s^−1^) reflects large variability in the genotype’s response. The most severe declines (>75%) were observed in IT-4, GR-4, and AdvL-4, showing a near collapse of C-assimilation (Table 2). Although less pronounced (50–75% P_n_ decline), PL-2, PL-4, IT-1, IT-3, and GR-3 were also greatly impacted by WL. A larger group of genotypes exhibited low (<25%, GR-1, AdvL-1, and Austrl-2) or moderate decreases (25–50%, PL-1, PL-3, PL-5, IT-2, GR-2, AdvL-5, Austrl-1, and Austrl-3) (Table 2). Finally, in sharp contrast, AdvL-2, AdvL-3, Austrl-4, and Austrl-5 plants showed no WL impact on P_n_.

Under WW conditions, stomatal conductance to water vapor (g_s_) values also showed strong genotypic variation (110–556 mmol H_2_O m^−2^ s^−1^) (Table 2). WL imposition clearly impacted g_s_, with a genotype-dependent reduction, often exceeding 50% relative to WW plants. The strongest declines were observed in several genotypes, including PL-3, PL-5, IT-1, IT-3, IT-4, GR-3, GR-4, AdvL-4, Austrl-1, and Austrl-3, where g_s_ dropped to very low values (32–72 mmol m^−2^ s^−1^). In contrast, some genotypes maintained (PL-4 and AdvL-1) or increased (AdvL-2, AdvL-3, Austrl-4, and Austrl-5) g_s_ under WL.

Overall, at T14, genotypes exhibited contrasting leaf gas exchange responses to waterlogging, expressed relative to their well-watered (WW) controls. Based on the coordinated responses of net photosynthetic rate (P_n_) and stomatal conductance (g_s_), distinct response patterns were identified. The most common pattern was a simultaneous reduction in both parameters, observed in PL-1, PL-2, PL-3, PL-5, IT-1, IT-2, IT-3, IT-4, GR-1, GR-2, GR-3, GR-4, AdvL-4, AdvL-5, Austrl-1, Austrl-2, and Austrl-3. This was followed by a pattern characterized by stable P_n_ and increased g_s_, found in AdvL-2, AdvL-3, Austrl-4, and Austrl-5. Two genotypes (PL-4 and AdvL-1) exhibited reduced P_n_ while maintaining stable g_s_ (Table 2). Across genotypes, changes in net photosynthetic rate (P_n_) were positively correlated with changes in stomatal conductance (g^s^) under waterlogging (Pearson r = 0.73, *p* < 0.001; *n* = 23), indicating that genotypic variation in photosynthetic responses was closely associated with variation in stomatal regulation. This relationship reflects the overall tendency for simultaneous reductions in P_n_ and g_s_ observed in most genotypes, although deviations were observed in genotypes maintaining or increasing g_s_ under WL.

### 2.5. Integrated Analysis of Genotypic Responses

**Figure 7:**
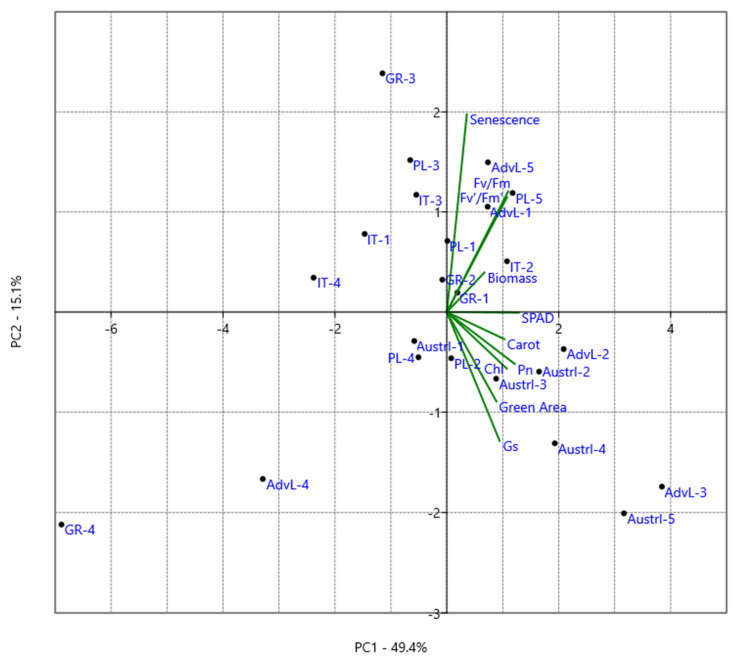
Principal component analysis (PCA) of physiological, pigment-related, and morphological traits measured in *T. aestivum* L. genotypes subjected to well-watered (WW) and waterlogging (WL) conditions during the tillering stage. The first two principal components (PC1 and PC2) explained 49.4% and 15.1% of the total variance, respectively. Variables included SPAD, total chlorophyll (Chl) and carotenoid (Carot) contents, net photosynthesis rate (P_n_), stomatal conductance to water vapor (g_s_), maximal (F_v_/F_m_) and actual (F_v_′/F_m_′) photochemical efficiency of photosystem II, green leaf area (green area), plant foliar biomass (biomass), and senescent foliar biomass (senescence). Traits are represented as vectors (green lines) and genotypes as points (black dots). The analysis included genotypes from five germplasm groups with distinct genetic backgrounds: Portuguese landraces (PL-1 to PL-5), varieties with the introduction of Italian germplasm (IT-1 to IT-4), Post-Green Revolution varieties with CIMMYT germplasm (GR-1 to GR-4), advanced lines (AdvL-1 to AdvL-5) from the Portuguese Cereal Breeding Programme, and Australian varieties (Austrl-1 to Austrl-5).

To allow an integrated view of the above-reported responses, a principal component analysis (PCA) was performed using selected physiological, pigment-related, and morphological traits associated with the maintenance of photosynthetic function and green leaf area, chosen based on their biological relevance and to avoid redundancy among highly correlated variables (Figure 7).

The first two principal components explained a substantial proportion (64.5%) of the total variance, allowing clear discrimination of genotypes according to their performance under WL stress. PC1 accounted for 49.4% of the total variance and was strongly associated with photosynthetic performance and leaf functional integrity, showing high positive loadings (0.30–0.41) for SPAD, total chlorophyll and carotenoid contents, P_n_, g_s_, F_v_/F_m_, and F_v_′/F_m_′. This component represents an integrated photosynthetic axis reflecting the ability to maintain an active photosynthetic apparatus under waterlogging stress (Figure 7). Genotypes with high PC1 scores were characterized by higher pigment content, enhanced photochemical efficiency, and greater carbon assimilation capacity. Accordingly, genotypes differed markedly in their overall tolerance to WL. AdvL-3, Austrl-5, and AdvL-2 exhibited the highest PC1 scores and were, therefore, identified as the most tolerant genotypes, whereas GR-4, AdvL-4, and IT-4 showed the lowest scores, indicating greater sensitivity to WL. A complete ranking of genotypes according to PC1 scores is provided in Appendix A, Table A1.

The second principal component (PC2) explained 15.1% of the total variance and was mainly associated with senescence, driven by the strong contribution of senescent biomass (Loading = 0.63). This axis reflects variation in stress-induced tissue degradation. The PCA biplot showed a clear clustering pattern, with genotypes exhibiting higher photosynthetic performance on the positive side of PC1, whereas those on the negative side showed the opposite response, pointing to higher tolerance or sensitivity to WL, respectively.

**Figure 8:**
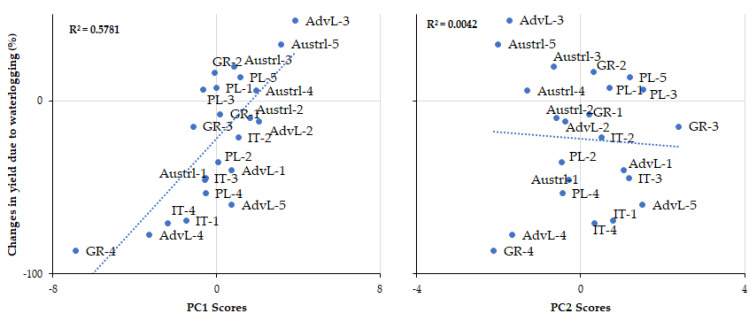
Relationships between principal component scores (PC1 and PC2) derived from PCA of physiological, pigment, and morphological traits and relative yield variation under waterlogging in *T. aestivum* L. genotypes. PC1 scores are shown in the left panel and PC2 scores in the right panel. Yield data were obtained from previously published results under the same experimental conditions and are not presented here. Each point represents an individual genotype from five germplasm groups with distinct genetic backgrounds: Portuguese landraces (PL-1 to PL-5), varieties with introduced Italian germplasm (IT-1 to IT-4), Post-Green Revolution varieties with CIMMYT germplasm (GR-1 to GR-4), advanced lines (AdvL-1 to AdvL-5) from the Portuguese Cereal Breeding Programme, and Australian varieties (Austrl-1 to Austrl-5). The dotted lines represent the regression (trend) lines corresponding to the coefficient of determination (R^2^) indicated for each relationship.

To further assess the biological relevance of the PCA, genotype scores on PC1 were used as an integrated proxy of plant physiological performance and related to yield variation under WL (Figure 8). As yield represents the ultimate agronomic outcome of stress responses, this approach allowed for an evaluation of whether early physiological traits assessed at the tillering stage are associated with final productivity under WL. Although yield responses under WL are not presented in this study, previously published data [30] obtained under the same experimental conditions were used solely to evaluate the relationship between PCA-derived components and agronomic performance (Figure 8).

A strong positive relationship was observed between PC1 scores and yield variation due to WL (R^2^ = 0.5781), indicating that PC1 captures key determinants of productivity under WL. Genotypes with higher PC1 scores exhibited improved yield performance, whereas those with lower scores showed substantial yield reductions (Figure 8). In contrast, PC2 showed no meaningful relationship with yield variation (R^2^ = 0.0042), suggesting that this component, mainly associated with senescence-related variation, does not contribute significantly to agronomic performance in this dataset (Figure 8).

## 3. Discussion

The present study found substantial genotypic variability in both morphological and physiological traits during waterlogging at the tillering stage. This variability was observed across groups representing different breeding stages, indicating that selection has not eroded genotypic diversity. Including these groups strengthens the robustness and relevance of the present study for breeding programs focused on stress resilience.

Beyond morphological responses, the present results highlight that tolerance to waterlogging is not solely associated with the maintenance of green biomass but also with the preservation of functionally active green tissue capable of sustaining photosynthesis. In our study, genotypes maintaining photosynthetic rates and photochemical efficiency, as well as higher PC1 scores, performed better under WL (e.g., AdvL-3, Austrl-5, Austrl-4), whereas others retained green leaf area despite marked physiological impairment (e.g., PL-2, Austrl-1). This suggests that the ability to maintain an active photosynthetic apparatus and canopy functionality is an important component of tolerance, potentially more relevant than the mere retention of green leaf area. However, waterlogging tolerance is a complex trait likely involving additional physiological and morphological mechanisms not addressed in this study. Together, these findings provide a framework for interpreting the contrasting responses observed among genotypes. Consistent with this, changes in the elongation rate of the main culm (Figure 1) reflected contrasting growth patterns among genotypes under WL. Decreases were observed across all germplasm groups, except the Australian genotypes, whereas increases occurred in four genotypes (Austrl-1, Austrl-2, Austrl-4, and Austrl-5), as well as in one advanced line (AdvL-2), with the latter interestingly resulting from a cross between Italian and Australian germplasm. Overall, genotypes exhibited diverse responses, including growth inhibition, maintenance, or stimulation. These patterns are consistent with previous studies reporting reduced plant height under waterlogging due to energy limitations associated with anaerobic metabolism [31,32,33], as well as elongation responses associated with escape strategies under excess water [12,34]. However, under WL conditions, where the aerial part is not submerged, such elongation responses, often mediated by ethylene accumulation, do not necessarily confer an adaptive advantage. Notably, in the genotypes exhibiting enhanced main culm elongation, this response was not consistently accompanied by proportional increases in leaf biomass accumulation, indicating that stem extension and leaf growth may be regulated independently under waterlogging. This suggests that increased elongation may reflect altered growth regulation and developmental dynamics rather than enhanced vegetative biomass production [11,34,35,36].

Waterlogging also contrastingly impacted plant total foliar biomass, further highlighting genotypic differences in growth responses. Across the 23 genotypes, total biomass decreased in five and increased in four, while remaining stable in the majority (Figure 2). These changes were primarily driven by variations in tiller biomass, which accounted for most of the observed decreases and increases, whereas changes in the main culm were less frequent and generally occurred in combination with tiller responses. Overall, these contrasting responses suggest a degree of growth plasticity that may contribute to maintaining canopy structure under adverse conditions, partially buffering stress effects, although this does not necessarily translate into stable yield [30,37,38,39]. Reductions in tiller biomass under WL have been widely reported and are commonly associated with decreased tiller initiation and/or impaired development of surviving tillers [12,30,37,40,41,42,43,44]. The sensitivity of tillers observed in PL-3, GR-2, GR-4, and AdvL-4 is consistent with previous studies in wheat and other cereals, where limited oxygen availability restricts the growth and development of secondary shoots [12,37,42,43,45,46]. In contrast, the increase in tiller biomass observed in PL-5, AdvL-1, AdvL-3, AdvL-5, Austrl-2, and Austrl-4 may reflect compensatory growth responses that partially offset stress-induced damage, as is the case of premature tissue senescence [39,47].

Since total biomass comprises both green and senescent leaves, increased plant biomass does not necessarily indicate tolerance, as part of this biomass may correspond to senescent tissue. This is particularly relevant for tillers, which may function as transient reservoirs of assimilates, with remobilization of carbon and nutrients toward the main culm to sustain its growth. This process may also involve the reallocation of resources from later-formed tillers to primary shoots, contributing to the maintenance of morphological integrity under waterlogging [38] but potentially leading to the early senescence of newly formed tillers. Simultaneous enhancement of senescence in both organs indicates a more generalized impairment of plant growth, as observed in highly sensitive genotypes, such as GR-4, reflecting a reduced capacity to maintain functional leaf area and canopy integrity, which ultimately compromises resource acquisition and overall plant performance under waterlogging stress [48,49].

These morphological responses, particularly the balance between biomass production and senescence, have direct implications for canopy organization under stress (Figure 3). In this context, a pronounced genotypic variability was observed in the capacity to maintain green leaf area under waterlogging, ranging from genotypes exhibiting only marginal increases in senescence to others reaching very high levels by the end of the stress period. Notably, several genotypes displayed stable total biomass despite concomitant increases in senescence and reductions in green leaf area. Thus, although plant biomass accumulation has been considered an intuitive indicator of stress tolerance [50,51,52], the present results indicate that apparent morphological stability at the whole plant level may not capture substantial internal shifts in tissue composition and function. In fact, the apparent stability of total biomass and green leaf area observed in PL-2, Austrl-1, and Austrl-3 could be interpreted as indicative of tolerance, in line with previous findings [27,51,52]; yet, our results point that this apparent stability did not fully capture the underlying canopy dynamics, as distinct patterns of decline, maintenance, or increases of green area among tillers and/or the main culm were observed. Among these three genotypes, however, Austrl-3 emerged as the only one that consistently maintained both total biomass and green leaf area at the whole plant level, as well as within both the main culm and tillers simultaneously, indicating a more coordinated structural stability under stress conditions. Overall, these findings suggest that morphological tolerance to waterlogging is largely associated with the capacity to maintain tiller integrity, sustain or compensate through tillering, and delay senescence.

Importantly, total leaf biomass does not provide information on the amount of photosynthetically active tissue, as it integrates both green and senescent fractions. In this context, green leaf area emerges as a more informative morphological indicator, as it better captures the active canopy fraction. Accordingly, reductions in green leaf area provide a more sensitive indication of morphological impairment than total biomass alone, especially when overall biomass remains unchanged despite a significant accumulation of senescent leaves. A larger green leaf area is commonly used as a proxy for enhanced plant C-assimilation capacity. However, this morphological trait alone does not necessarily translate into proportional functional performance, as the physiological status of the remaining photosynthetically active tissue can vary substantially under stress. Under WL conditions, reductions in photosynthetic capacity are frequently driven not only by changes in leaf green area but also by coordinated impairments in stomatal and non-stomatal processes, including limitations in CO_2_ diffusion, chlorophyll degradation, and dysfunction of the photosynthetic apparatus [53,54,55]. This supports a partial decoupling between morphological and functional traits, whereby plant performance is influenced by both the extent of green tissue and its intrinsic physiological integrity, rather than by morphological attributes alone [12]. In this context, chlorophyll-related traits, such as SPAD values (Figure 5), provide additional insight into canopy physiological status, as they reflect variation in leaf pigment content and are commonly associated with photosynthetic capacity and nitrogen status [53,56,57]. In line with this, SPAD values decreased across all genotypes under WL (Figure 5), although the magnitude of reduction varied substantially, being particularly pronounced in PL-4, IT-1, IT-4, GR-3, GR-2, GR-4, AdvL-4, Austrl-1, and Austrl-3. This overall decline reflects a generalized reduction in chlorophyll content under stress, yet the genotypic differences in its extent further support the variability in physiological sensitivity to WL. Moreover, as a non-destructive and rapid method, SPAD measurements enable repeated assessments on the same plants over time, allowing a more integrative evaluation of canopy dynamics under stress. However, SPAD represents a bulk estimate of chlorophyll content and, therefore, does not explicitly capture spatial heterogeneity in pigment distribution or functional status within the canopy. This limitation is particularly relevant under WL stress, where senescence is spatially structured and progresses differentially along the culm, leading to strong intra-plant variability in leaf greenness and physiological performance [56]. Thus, as the values presented in this study correspond to averages across all leaves of the main culm, their interpretation requires caution, particularly under conditions that promote position-dependent senescence [1,53]. Because waterlogging promotes basal leaf senescence, average SPAD values may underestimate variability among leaves of different ages and positions, potentially masking genotype-specific responses. In fact, whereas some genotypes may exhibit a generalized decline in chlorophyll content across the culm, others were able to maintain chlorophyll levels in younger leaves with values comparable to those observed under WW conditions, despite overall reductions at the culm level. This indicates a degree of functional prioritization within the canopy, whereby senescence progresses in older leaves while photosynthetic competence is partially preserved in recently developed leaves. Pigment quantification by spectrophotometry was performed on a defined leaf position (the second leaf from the top of the main culm), providing a more localized assessment of chlorophyll content (Figure 6). Therefore, while SPAD offers an integrative canopy-level proxy, direct pigment measurements allow a more precise evaluation of the physiological status of specific leaves, and the two approaches provide information at different spatial and organizational scales.

Overall, reductions in chlorophyll and carotenoid contents were observed across most of the genotypes, indicating a general decline in light-harvesting and photoprotective capacity under hypoxic conditions [27,58,59]. However, the magnitude of these changes varied markedly among genotypes, highlighting that the functional competence of the remaining green tissue can differ substantially, even when green leaf area is partially maintained. The most pronounced pigment degradation was observed in GR-4, with chlorophyll declining by ca. 82%, accompanied by strong reductions (ca. 74%) in carotenoid content, indicating severe impairment of the photosynthetic apparatus. Similar, although less extreme, responses were observed in other sensitive genotypes. In contrast, genotypes such as AdvL-2, AdvL-3, and Austrl-5 maintained both chlorophyll and carotenoid levels, suggesting a greater capacity to preserve photosynthetically active tissue under stress. Notably, Austrl-3 was the only genotype showing an increase in carotenoid content while simultaneously maintaining chlorophyll levels. This response, combined with the absence of changes in relative water content (RWC) and green leaf area, indicates an enhanced ability to sustain tissue hydration, structural integrity, and photoprotective capacity under stress. Chlorophyll degradation implies a decreased capacity for light harvesting and carbon assimilation [60] and is a well-established indicator of stress-induced senescence, consistent with patterns reported under waterlogging and drought [23,60,61]. In contrast, the maintenance of chlorophyll and carotenoid contents reflects the ability to preserve photosynthetically active tissue under adverse conditions. This is particularly relevant for carotenoids, given their role in photoprotection [62,63,64]. Differences in carotenoid content further reflect variation in the dissipation of excess excitation energy and the prevention of oxidative damage; their maintenance, or even increase, indicates a more effective protective response, whereas their depletion suggests increased susceptibility to oxidative stress. These responses support the view that pigment degradation and impaired photoprotection may precede declines in photosynthetic efficiency under stress.

The ability to maintain leaf hydration under waterlogging, when plants are exposed to a hypoxic rhizosphere, is critical for preserving morphological and cellular integrity as well as sustaining photosynthetic performance. While most genotypes maintained high RWC in the main culm (Figure 4), a subset (PL-4, IT-1, GR-4; AdvL-2, and AdvL-4) exhibited marked reductions that coincided with losses in green leaf area, suggesting that decreased leaf hydration may have contributed to the loss of functional leaf structure. RWC decreases likely reflect limitations in root water uptake under hypoxia, associated with a shift to anaerobic metabolism that reduces energy availability, impairs root growth, and promotes root death [12,65]. In addition, hypoxia can impair aquaporin function, reducing hydraulic conductivity and limiting water transport to the shoots [66,67].

Stomatal regulation plays a central role in plant responses to waterlogging, as oxygen deprivation in the root zone rapidly affects water relations and leads to partial stomatal closure, ultimately restricting gas exchange and carbon assimilation [12,33,68,69]. In the present study, most genotypes (PL-1, PL-2, PL-3, PL-5, all IT and GR genotypes, AdvL-4, AdvL-5, Austrl-1, Austrl-2, and Austrl-3) exhibited concurrent reductions in P_n_ and g_s_ (Table 2), suggesting that stomatal limitation was a major factor constraining photosynthetic performance under waterlogging conditions. This coordinated decline further supports a major role of stomatal limitations in the observed reduction in photosynthetic performance in these genotypes. In several cases, the decline in P_n_ and g_s_ was further accompanied by reduced F_v_/F_m_ and/or F_v_′/F_m_′, suggesting that PSII efficiency was also affected by waterlogging stress [70], indicating combined diffusional and photochemical limitations. In contrast, genotypes such as PL-4 and AdvL-4 exhibited reduced P_n_ without marked changes in g_s_ or chlorophyll fluorescence parameters, suggesting that factors other than stomatal regulation contributed to the observed decline in photosynthetic performance. Under these conditions, biochemical constraints affecting carbon assimilation may have played a role. However, the specific mechanisms underlying these responses cannot be determined from the present data [33,71,72].

Chlorophyll fluorescence responses further highlighted genotypic differences in photochemical behavior. Reductions in F_v_/F_m_ and/or F_v_′/F_m_′ observed in several genotypes indicate PSII impairment under stress [70,73]. In some genotypes, these reductions occurred alongside decreases in g_s_ and P_n_, reinforcing the co-occurrence of stomatal and photochemical limitations. Conversely, some genotypes maintained relatively stable fluorescence parameters despite reductions in P_n_, indicating partial decoupling between photochemistry and carbon assimilation [72,73].

A subset of genotypes, including AdvL-2, AdvL-3, Austrl-4, and Austrl-5, maintained stable P_n_ under WL, and this stability was concomitant with increased g_s_. In all four genotypes, this stability was associated with increased g_s_, suggesting an enhanced capacity to maintain gas exchange and sustain photosynthetic activity under stress conditions [12,68,69]. However, fluorescence responses varied among these genotypes, indicating that PSII stability was not uniform and suggesting different physiological adjustment strategies rather than a single tolerance mechanism.

Overall, the results show that waterlogging induces a complex interaction between stomatal regulation and photochemical performance, with substantial genotypic variation in the relative contribution of these processes. While reductions in g_s_ were closely associated with declines in photosynthetic performance in most genotypes, other physiological factors likely also contributed to the observed responses. Importantly, genotypes with stable P_n_ (AdvL-2, AdvL-3, Austrl-4, and Austrl-5) exhibited increased g_s_, reflecting a stronger capacity to maintain gas exchange under stress, although PSII responses were variable. This indicates that maintenance of P_n_ is not necessarily associated with full PSII stability but rather with an effective stomatal compensation strategy that partially offsets stress-induced limitations.

These findings highlight distinct physiological strategies underlying waterlogging responses in bread wheat, ranging from strong stomatal sensitivity coupled with photochemical impairment to more stable photosynthetic performance supported by enhanced stomatal regulation and variable PSII efficiency.

The multivariate analysis reinforced the patterns observed in individual traits, highlighting that genotypic variation in waterlogging tolerance is largely determined by the coordinated maintenance of photosynthetic performance, leaf functional integrity, and pigment content. The first principal component, representing an integrated photosynthetic axis, separated tolerant and sensitive genotypes, confirming that the ability to sustain chlorophyll and carotenoid levels, photochemical efficiency, and carbon assimilation under stress is central to tolerance. In contrast, the second component reflected variation in senescence, emphasizing that stress-induced tissue degradation contributes to the reduction in functional leaf area and overall photosynthetic capacity. These patterns illustrate that tolerance is not dictated by single traits but emerges from the interaction of multiple morphological and functional mechanisms.

The strong relationship between PC1 (associated with photosynthetic traits) and yield performance under WL underscores the agronomic relevance of maintaining photosynthetic function and green leaf area. Together, these results suggest that selection for genotypes capable of preserving coordinated physiological performance during early vegetative stages is likely to improve productivity in waterlogging-prone environments. These findings have direct implications for breeding strategies, highlighting that selection based on isolated morphological traits may be insufficient to capture stress tolerance. Instead, integrated indicators combining chlorophyll fluorescence and photosynthetic performance emerge as robust tools to identify genotypes capable of maintaining functional canopy activity under WL.

Overall, this study demonstrates that waterlogging tolerance at the tillering stage, a key early vegetative growth phase, is primarily driven by the capacity to sustain coordinated physiological function rather than by morphological traits alone. This functional perspective provides a robust framework for early-stage screening and supports the development of wheat genotypes better adapted to increasingly waterlogging-prone environments.

## 4. Materials and Methods

### 4.1. Plant Material

The studied bread wheat (*T. aestivum* L.) germplasm was supplied by the Portuguese Cereal Breeding Programme (PCBP) taking place at the National Institute of Agricultural and Veterinary Research, I.P. (INIAV), and includes Australian varieties and genotypes from different stages of wheat cultivation in Portugal (Table 3).

### 4.2. Experimental Design

#### 4.2.1. Seed Multiplication

To ensure adequate seed vigor and uniform germination capacity, certified seeds of each *T. aestivum L*. genotype were multiplied in growth chambers (Fitoclima 10000 EHHF, ARALAB, Rio de Mouro, Portugal) under controlled conditions of temperature (22/15 °C, day/night), irradiance (ca. 500–600 μmol m^−2^ s^−1^), relative humidity (70/75%, day/night), photoperiod (14 h), and CO_2_ (400 μL L^−1^) in 5 L pots with field-collected loamy clay soil, as previously described [11].

#### 4.2.2. Plant Establishment and Growth Conditions

**Figure A1:**
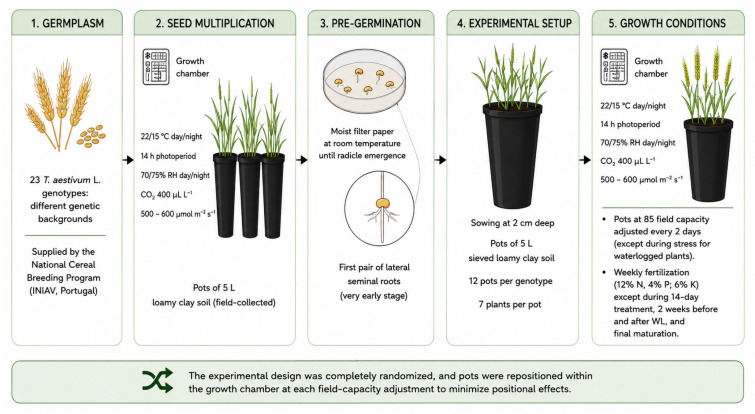
Schematic representation of the early stages of the experimental design adopted for the evaluation of 23 *Triticum aestivum* L. genotypes under controlled-environment conditions. The figure was originally designed by the authors and subsequently refined using AI-assisted graphical tools under full author supervision. Parts of the graphical elements were developed with the assistance of AI-based tools, using original experimental data and author-generated inputs. The authors performed the final design, validation, and assembly of all figure components.

**Figure A2:**
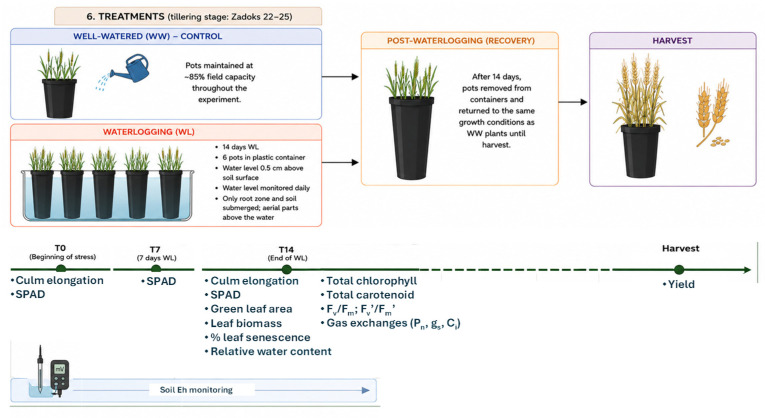
Experimental design of the waterlogging and post-waterlogging treatments and associated physiological, morphological, and biochemical evaluation scheme. The figure was originally designed by the authors and subsequently refined using AI-assisted graphical tools under full author supervision. Parts of the graphical elements were developed with the assistance of AI-based tools, using original experimental data and author-generated inputs. The authors performed the final design, validation, and assembly of all figure components.

**Figure A3:**
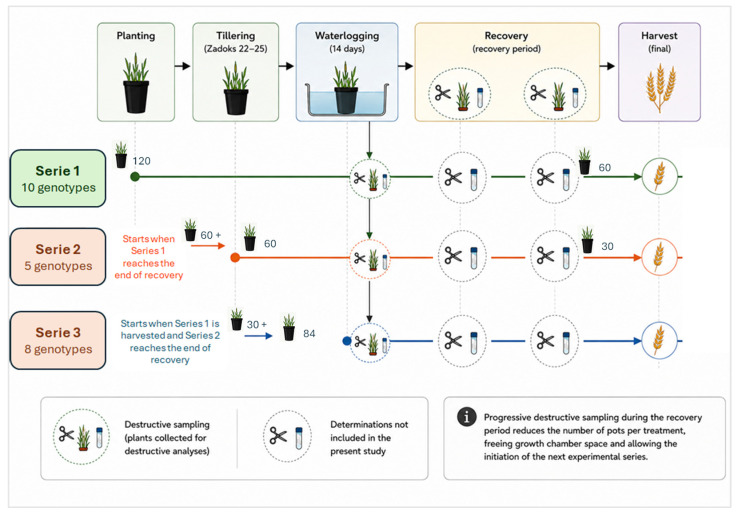
Timeline of the partially overlapping experimental series, illustrating destructive sampling events, pot reduction, and sequential use of growth chamber space throughout the experiment. The figure was originally designed by the authors and subsequently refined using AI-assisted graphical tools under full author supervision. Parts of the graphical elements were developed with the assistance of AI-based tools, using original experimental data and author-generated inputs. The authors performed the final design, validation, and assembly of all figure components.

A schematic overview of the experimental workflow is provided in Appendix B, Figure A1, Figure A2 and Figure A3.

The newly multiplied seeds were pre-germinated on moist filter paper at room temperature until the emergence of the radicle and the first pair of the lateral seminal root. Germinated seeds were sown at a 2 cm depth, with the germ end facing downward, in 5 L pots filled with sieved loamy clay soil. A total of 12 pots were established per genotype, with 7 plants per pot. Of these, 6 pots were assigned to the well-watered treatment (WW) and 6 pots to the waterlogging treatment (WL). Plants were grown in the same walk-in growth chambers used for seed multiplication under identical controlled-environmental conditions. Pots were maintained at ca. 85% field capacity (FC), adjusted every two days, except during the waterlogging period for WL plants. Plants were fertilized weekly with 250 mL of a commercial nutrient solution containing 12% N, 4% P, and 6% K (Complesal, Bayer, Ammerbuch, Germany), except during the WL period, the two weeks immediately before and after stress imposition, and the final maturation stage. The experimental design was completely randomized, and pots were repositioned within the growth chamber at each FC adjustment to minimize positional effects (Appendix B, Figure A1).

#### 4.2.3. Waterlogging Implementation

Treatments were imposed at the tillering stage (Zadoks growth stages 22–25; [74]), with WW plants maintained at ca. 85% FC and WL plants subjected to 14 days of waterlogging by placing six pots inside plastic containers filled with water until the water level reached ca. 0.5 cm above the soil surface. Water levels were monitored and carefully replenished when necessary, minimizing air entry into the substrate and assuring that only the root zone and soil were submerged, while the aerial plant parts remained above the water surface. After 14 days, pots were removed from the containers and returned to the same growth conditions as WW plants until harvest. Some measurements were performed at T0 (SPAD, main culm elongation), T7 (SPAD), and T14 (SPAD, main culm elongation). All remaining physiological, morphological, and biochemical evaluations included in this manuscript were performed at T14. Additional analyses not included in the present study were also conducted during the experimental period and required destructive plant sampling. Consequently, at final harvest, only 3 pots with 3 plants each per genotype and treatment remained available for analysis (Appendix B, Figure A2).

#### 4.2.4. Experimental Series

Due to growth chamber space limitations, the experiment was conducted in three partially overlapping experimental series.

Serie 1: PL-2, PL-4, PL-5, IT-2, AdvL-1, AdvL-2, AdvL-3, AdvL-5, Austrl-4, and Austrl-5; Series 2: IT-1, GR-2, Austrl-1, Austrl-2, and Austrl-3; Series 3: PL-1, PL-3, IT-3, IT-4, GR-1, GR-3, GR-4, and AdvL-4.

Growth chamber space was progressively released through destructive sampling, allowing partially overlapping experimental series. All series were conducted under strictly controlled and identical environmental conditions and standardized growth protocols to ensure consistency throughout the study. Temperature, relative humidity, photoperiod, light intensity, irrigation management, and fertilization regimes were maintained constant across all series. To minimize potential batch effects, genotypes were distributed across experimental series without systematic association between genotype groups. Within each series, the experimental design was completely randomized at the pot level, and pots were regularly repositioned within the growth chamber to minimize positional effects. This experimental structure ensured full comparability across series despite their partial temporal overlap (Appendix B, Figure A3).

### 4.3. Soil Reduction–Oxidation Potential

Soil redox potential (Eh) was assessed at the onset of WL (T0), after 24 h (T1), and 7 days of WL (T7), and at the end of the stress period (T14). Measurements were performed with a portable Eh meter (ORP-5, XS Instruments, Carpi, Italy) in two pots per genotype and treatment. Readings were taken at two locations within each pot, one at the center and another at an intermediate position between the center and the pot edge, both at a depth of 6 cm. Preliminary measurements at additional equidistant points did not reveal any significant spatial variation in Eh values; therefore, two measurement points were considered representative, and values were recorded after signal stabilization. The mean of the two measurements was calculated for each pot and considered as a biological replicate for statistical analysis. The data from all experimental series were combined for subsequent analyses, as no significant differences were identified among the series.

### 4.4. Measurement of Elongation Rate of the Main Culm

The height of the main culm (cm) was measured at the end of the waterlogging period in three distinct pots (three plants per pot) for each treatment and genotype. The average daily elongation rate values were calculated as the difference between the main culm height at the end and at the beginning of the stress period, divided by the number of days from that period (14) and expressed as cm day^−1^.

### 4.5. Leaf Area, Leaf Biomass, and Senescence

At the end of the waterlogging period (T14), one plant from three distinct pots per treatment and genotype was collected. The leaves were individually detached from the stem for each culm (main culm and tillers).

The surface area of the green leaves was assessed for all genotypes in both treatments using a foliar area meter LI-3000A (LI-Cor Inc., Lincoln, NE, USA).

Leaves were then separated into green and chlorotic fractions within each culm, oven-dried at 80 °C until constant weight, cooled in a desiccator, and weighed. Total foliar biomass was calculated as the sum of green and senescent leaf dry weight (g DW), and senescence was expressed as the percentage of total foliar biomass.

### 4.6. Leaf Relative Water Content

The plant’s water status, expressed as the relative water content (RWC), was estimated at the end of the waterlogging period (T14) following [68]. Briefly, three leaf discs (0.35 cm^2^ each) were collected from the second fully expanded leaf of the main culm of four plants per pot (12 discs per pot). Three pots per treatment and genotype were used. Fresh weight (FW) was assessed immediately after sampling, turgid weight (TW) was determined after overnight rehydration in a humid chamber at 25 °C, and dry weight (DW) was obtained after drying at 80 °C for 48 h. RWC (%) was calculated as:

(1) $$RWC=\frac{FW-DW}{TW-DW}\times 100$$

### 4.7. Leaf Gas Exchanges

Leaf gas exchanges, including net photosynthetic rate (P_n_) and stomatal conductance to water vapor (g_s_), were measured using a portable CO_2_/H_2_O infrared gas analyzer exchange system LI-6400 (LI-Cor Inc., USA), with an external CO_2_ supply of ca. 500 μL L^−1^ and artificial LED irradiation of 500 μmol m^−2^ s^−1^ at 25 °C. Measurements were conducted at the end of the waterlogging period (T14), under photosynthetic steady-state conditions, after 2 h of light exposure, on the second top leaf of the main culm, in three plants per pot. Five pots per treatment and genotype were used.

### 4.8. Photochemical Efficiency of Photosystem II

Chlorophyll a fluorescence measurements were performed on the same leaves used for gas exchange using a FluorPen FP110/D (PSI, Drásov, Czech Republic). The maximal photochemical efficiency of PSII (F_v_/F_m_) was determined on predawn dark-adapted leaves after overnight dark adaptation. The actual photochemical efficiency of PSII (F_v_′/F_m_′) was measured on light-adapted leaves following 2 h of exposure to growth chamber irradiance using the QY protocol. The actinic light intensity during measurements was set to 550 μmol photons m^−2^ s^−1^, corresponding to the irradiance used under growth conditions. These measurements were used to assess the photochemical performance of PSII under control and waterlogging conditions.

### 4.9. SPAD Measurements

The relative chlorophyll content of all leaves from the main culm was assessed using a SPAD-502 Plus chlorophyll meter (Konica Minolta, Tokyo, Japan). Measurements were performed on three plants per pot, in all pots (six per treatment), for each genotype at the beginning (T0), after 7 days (T7), and at the end of the waterlogging period (T14). The results are expressed as the average SPAD value per main culm. As a non-destructive method, SPAD measurements enabled repeated assessments on all leaves of the main culm in the same plants over time.

### 4.10. Total Leaf Chlorophyll and Carotenoid Contents

Total chlorophylls and carotenoids were determined at the end of the stress period (T14) and extracted from pooled samples of the top two leaves of the main culm from four plants per pot. Three pots per treatment and genotype were used. For each replicate, 50 mg of fresh weight (FW) were placed in vials containing 10 mL of 100% methanol and stored at 4 °C in the dark for 72 h, according to [22]. Absorbance of the extracts was subsequently measured spectrophotometrically (Shimadzu UV160A, Kyoto, Japan). Pigment concentrations were calculated according to the equations for 100% methanol described by Lichtenthaler [75].

### 4.11. Statistical Analysis

A one-way or two-way ANOVA was used to assess the effects of water treatment and/or tissue (main culm or tillers), including their interaction, followed by Tukey’s HSD test for mean comparisons. Analyses were performed independently for each genotype using biological replicates (*n* = 3 to 6 pots). A significance level of 0.05 was adopted for all tests.

Principal component analysis (PCA) aimed to explore the relationships among physiological and morphological traits under waterlogging (relative to the percentage changes of the respective control, WW) and to identify the main sources of genotypic variation.

All data were analyzed using the software PAST (Paleontological Statistics software, version 3, University of Oslo, Norway).

## 5. Conclusions

Waterlogging tolerance in bread wheat at the tillering stage is primarily associated with the ability to maintain both green leaf area and photosynthetic function rather than morphological stability alone. Although substantial genotypic variability was observed across traits, tolerance was consistently associated with the ability to sustain photochemical efficiency and gas exchange under stress conditions.

Morphological traits such as biomass and green leaf area were not always reliable predictors of physiological performance, as several genotypes maintained canopy structure while exhibiting marked reductions in photosynthetic activity. In contrast, tolerant genotypes showed a more coordinated maintenance of photosynthetic performance and pigment-related traits under waterlogging, whereas sensitive genotypes displayed strong physiological impairment and accelerated senescence.

Multivariate analysis confirmed that an integrated photosynthetic trait axis was the main determinant of genotypic performance under waterlogging and was strongly associated with plant performance under stress, while senescence-related variation represented a secondary component of the response. Overall, these findings highlight that selection for waterlogging tolerance in wheat should prioritize functional physiological traits, particularly photosynthetic performance, rather than relying solely on morphological indicators.

## Figures and Tables

**Table 1:** Soil redox potential (Eh, mV) under well-watered (WW) and waterlogged (WL) conditions across the experimental period. Measurements were performed at the onset of the stress (T0), after 24 h (T1), and on days 7 (T7) and 14 (T14) of waterlogging, in two pots per genotype. Data are presented as means ± SE (*n* = 46 per treatment). Differences were assessed by two-way ANOVA followed by Tukey’s HSD test (*p* < 0.05). Different letters indicate significant differences among sampling times (T0, T1, T7, and T14) within each treatment (WW or WL), while * indicates significant differences between WW and WL at the same sampling time. The decline in Eh under WL reflects the progressive development of hypoxic/anoxic soil conditions.

	Days of Waterlogging			
Treatment	T0	T1	T7	T14
WW	402.7 ± 16.0 a *	410.6 ± 15.8 a *	392.5 ± 13.1 a *	388.4 ± 12.4 a *
WL	407.5 ± 20.5 a	126.4 ± 22.8 b	36.4 ± 8.8 c	−11.9 ± 9.5 c

**Table 2:** Changes in maximal (F_v_/F_m,_ dark adapted) and actual (F_v_′/F_m_′, photosynthetic steady-state) photochemical efficiency of photosystem II, as well as leaf gas exchange parameters (net photosynthesis, P_n_; stomatal conductance to water vapor, g_s_), in *T. aestivum* L. genotypes from five germplasm groups with distinct genetic backgrounds: Portuguese landraces (PL), varieties with introduced Italian germplasm (IT), Post-Green Revolution varieties with CIMMYT germplasm (GR), advanced lines (AdvL) from the Portuguese Cereal Breeding Programme, and Australian varieties (Austrl). Measurements were performed in well-watered (WW) and waterlogged (WL) plants on the second top leaf of the main culm at the end of the 14-day experiment. Statistical analysis was performed by one-way ANOVA followed by Tukey’s HSD test (*n* = 3–6, *p* < 0.05). * Indicates differences between WW and WL for each genotype and parameter, placed above the higher mean value.

	F_v_/F_m_		F_v_′/F_m_′		P_n_		g_s_	
					(µmol CO_2_ m^−2^ s^−1^)		(mmol H_2_O m^−2^ s^−1^)	
	WW	WL	WW	WL	WW	WL	WW	WL
PL-1	0.818 ± 0.002 *	0.796 ± 0.002	0.604 ± 0.017	0.572 ± 0.001	16.9 ± 0.8 *	10.5 ± 0.7	455 ± 60 *	250 ± 45
PL-2	0.806 ± 0.004 *	0.775 ± 0.010	0.595 ± 0.018	0.537 ± 0.043	19.0 ± 0.4 *	8.8 ± 0.8	256 ± 22 *	136 ± 22
PL-3	0.803 ± 0.001	0.805 ± 0.003	0.581 ± 0.026	0.556 ± 0.021	11.2 ± 0.5 *	7.0 ± 0.4	165 ± 44 *	55 ± 4
PL-4	0.815 ± 0.012	0.807 ± 0.004	0.591 ± 0.011	0.616 ± 0.028	20.6 ± 0.9 *	10.2 ± 0.4	110 ± 12	119 ± 7
PL-5	0.820 ± 0.006	0.794 ± 0.011	0.619 ± 0.019	0.573 ± 0.011	18.1 ± 1.3 *	11.3 ± 0.8	247 ± 48 *	68 ± 7
IT-1	0.802 ± 0.002 *	0.737 ± 0.025	0.577 ± 0.016	0.482 ± 0.049	18.1 ± 1.2 *	5.8 ± 2.1	332 ± 30 *	68 ± 20
IT-2	0.817 ± 0.001	0.802 ± 0.006	0.603 ± 0.023	0.611 ± 0.018	18.1 ± 0.1 *	12.8 ± 1.2	500 ± 67 *	190 ± 41
IT-3	0.810 ± 0.006	0.798 ± 0.010	0.596 ± 0.010	0.631 ± 0.002 *	17.4 ± 0.7 *	6.6 ± 1.8	453 ± 33 *	72 ± 18
IT-4	0.805 ± 0.003 *	0.772 ± 0.008	0.594 ± 0.012	0.563 ± 0.015	17.6 ± 0.5 *	2.9 ± 0.7	313 ± 32 *	37 ± 4
GR-1	0.822 ± 0.002	0.810 ± 0.005	0.567 ± 0.005	0.604 ± 0.015	20.5 ± 0.4 *	16.0 ± 1.0	556 ± 52 *	250 ± 65
GR-2	0.820 ± 0.000	0.821 ± 0.002	0.595 ± 0.013	0.605 ± 0.002	18.1 ± 1.1 *	9.1 ± 1.8	331 ± 95 *	107 ± 24
GR-3	0.819 ± 0.001	0.807 ± 0.006	0.561 ± 0.008	0.611 ± 0.012 *	20.2 ± 0.9 *	7.5 ± 0.9	402 ± 74 *	83 ± 12
GR-4	0.804 ± 0.005 *	0.229 ± 0.002	0.525 ± 0.005 *	0.103 ± 0.052	15.1 ± 1.3 *	3.3 ± 0.4	368 ± 55 *	48 ± 7
AdvL-1	0.814 ± 0.006	0.792 ± 0.007	0.565 ± 0.009	0.570 ± 0.015	19.4 ± 0.4 *	15.5 ± 1.5	200 ± 14	126 ± 54
AdvL-2	0.824 ± 0.001	0.813 ± 0.005	0.548 ± 0.019	0.559 ± 0.026	20.9 ± 0.7	20.5 ± 0.6	381 ± 13	499 ± 66 *
AdvL-3	0.822 ± 0.003 *	0.810 ± 0.002	0.593 ± 0.012	0.599 ± 0.012	20.0 ± 1.1	19.9 ± 2.5	163 ± 29	537 ± 50 *
AdvL-4	0.815 ± 0.004 *	0.528 ± 0.042	0.597 ± 0.021 *	0.259 ± 0.043	14.7 ± 0.8 *	2.5 ± 0.7	367 ± 30 *	33 ± 6
AdvL-5	0.803 ± 0.007	0.784 ± 0.012	0.554 ± 0.024	0.548 ± 0.037	21.2 ± 1.5 *	15.2 ± 2.0	198 ± 51 *	94 ± 34
Austrl-1	0.799 ± 0.001	0.740 ± 0.024	0.541 ± 0.032	0.462 ± 0.080	11.2 ± 0.5 *	4.9 ± 0.2	242 ± 52 *	58 ± 3
Austrl-2	0.800 ± 0.004	0.790 ± 0.008	0.575 ± 0.026	0.615 ± 0.005	18.3 ± 0.1 *	14.7 ± 1.2	549 ± 26 *	360 ± 72
Austrl-3	0.798 ± 0.005 *	0.704 ± 0.025	0.605 ± 0.009 *	0.447 ± 0.017	11.3 ± 1.0 *	7.1 ± 1.2	242 ± 63 *	68 ± 14
Austrl-4	0.816 ± 0.002 *	0.806 ± 0.002	0.561 ± 0.0023	0.603 ± 0.015	21.1 ± 0.2	22.9 ± 0.4	259 ± 32	459 ± 41 *
Austrl-5	0.811 ± 0.001 *	0.803 ± 0.002	0.557 ± 0.009	0.585 ± 0.004 *	18.2 ± 0.3	19.3 ± 1.5	234 ± 21	442 ± 26 *

**Table 3:** Bread wheat (*T. aestivum* L.) germplasm. Genotypes supplied by the PCBP belonging to five groups according to genetic background.

Germplasm Group	Code	Genotype	ID
Portuguese landraces from Vasconcelos ancient collection (1933), which accurately reflected the genetic diversity of regional Portuguese wheat prior to modern breeding	PL	Alentejano Ardito Mocho Cabeçudo Mocho de Espiga Quadrada Mocho de Espiga Branca	PL-1
			PL-2
			PL-3
			PL-4
			PL-5
Varieties with introduced Italian germplasm Developed by the PCBP and released between 1950 and 1970	IT	Restauração Chaimite Mara Pirana	IT-1
			IT-2
			IT-3
			IT-4
Post-Green Revolution varieties with introduced CIMMYT germplasm Developed by the PCBP and released between 1980 and 1989	GR	Caia Nabão Roxo Mondego	GR-1
			GR-2
			GR-3
			GR-4
Advanced lines Obtained through the PCBP or exchanges with the Mexican programme CIMMYT	AdvL	Ducula/Gondo//Sokol ^1^ Katunga × (Centauro/Vega) ^2^ Kennedy × Roxo ^3^ KLDR/Pewit1//Milan/Ducula ^1^ GUS/3/Prl/Sara/Tsi/Vee#5/… ^1^	AdvL-1
			AdvL-2
			AdvL-3
			AdvL-4
			AdvL-5
Australian germplasm	Austrl	BT-Schomburgk Escalibur Sunvale Sunlin Trident	Austrl-1
			Austrl-2
			Austrl-3
			Austrl-4
			Austrl-5

^1^ CIMMYT material; ^2^ Australian × Italian; ^3^ Australian × Portuguese.

## Data Availability

The original contributions presented in this study are included in the article. Further inquiries can be directed to the corresponding author.

## Abbreviations

The following abbreviations are used in this manuscript:

AdvL	Advanced line
Austrl	Australian variety
gs	Stomatal conductance
GR	Post-Green Revolution varieties
IT	Italian germplasm varieties
MC	Main culm
PCA	Principal component analysis
PL	Portuguese landraces
Pn	Net photosynthetic rate
PSII	Photosystem II
RWC	Relative water content
SPAD	Soil plant analysis development (chlorophyll index)
T	Tillers
T0	Beginning of the experiment
T7	7 days of waterlogging
T14	14 days of waterlogging
TCar	Total carotenoids
TChl	Total chlorophylls
WL	Waterlogging
WW	Well-watered

## Appendix A

**Table A1:** Ranking of the 23 bread wheat (*Triticum aestivum* L.) genotypes according to PC1 scores obtained from the principal component analysis. Higher PC1 scores indicate greater tolerance to waterlogging. The genotypes belong to five germplasm groups with distinct genetic backgrounds: Portuguese landraces (PL), varieties incorporating Italian germplasm (IT), post-Green Revolution varieties containing CIMMYT germplasm (GR), advanced lines (AdvL) from the Portuguese Cereal Breeding Programme, and Australian varieties (Austrl).

Rank	Genotype	PC1 Score
1	AdvL-3	3.844
2	Austrl-5	3.165
3	AdvL-2	2.088
4	Austrl-4	1.929
5	Austrl-2	1.649
6	PL-5	1.175
7	IT-2	1.075
8	Austrl-3	0.881
9	AdvL-5	0.734
10	AdvL-1	0.729
11	GR-1	0.188
12	PL-2	0.078
13	PL-1	0.010
14	GR-2	−0.081
15	PL-4	−0.507
16	IT-3	−0.546
17	Austrl-1	−0.582
18	PL-3	−0.655
19	GR-3	−1.150
20	IT-1	−1.468
21	IT-4	−2.381
22	AdvL-4	−3.288
23	GR-4	−6.888
